# Recurrent Lower Abdominal Pain in Older Children and Adolescents: Revised Clinical Approach

**DOI:** 10.1100/tsw.2005.10

**Published:** 2005-01-28

**Authors:** Harohalli Shashidhar, Hatim A. Omar

**Affiliations:** ^1^ Department of Pediatrics, Division of Pediatric Gastroenterology and Nutrition, University of Kentucky Medical Center, Lexington, KY 40536, USA; ^2^ Section of Adolescent Medicine, University of Kentucky Medical Center, University of Kentucky Medical Center, Lexington, KY 40536, USA

**Keywords:** abdominal pain, children, adolescents, United States

## Abstract

Pelvic and lower abdominal pain constitutes more than 30% of the referrals to our adolescent medicine and gastroenterology clinics. These cases are responsible for numerous school absences and significant cost to the healthcare system. This article will review the most common causes of lower abdominal and pelvic pain in adolescents, diagnostic and management issues, as well as prevention.

## INTRODUCTION

Acute and recurrent lower abdominal and pelvic pain is a common symptom encountered in the pediatric office and by emergency room physicians. Acute pain may represent a life-threatening situation and demand an emergent evaluation and intervention, while an adolescent presenting with pain of a longer duration requires a different approach. Either situation can be challenging and calls for systematic and analytical clinical decision making. In this article, the clinical approach to an older child or adolescent presenting with lower abdominal and pelvic pain of a few days to a few weeks duration is presented. This discussion pertains primarily to nongynecologic diagnoses.

## HISTORY IS PARAMOUNT

> 16-year-old female presents with abdominal pain for 2–3 weeks duration. The pain is described as crampy and poorly localized. The pain is worse in the last 4 days and worse with eating. She has had nonbilious emesis since onset of the pain. She also reports watery stools without blood mucous once or twice a day in the last week. Prior history includes involuntary weight loss of 3.6 kg and fatigue, as well as a surgery for fissures and hemorrhoids at 13 years of age. She had experienced similar symptoms at the time including emesis, weight loss, and diarrhea. She describes a heavy menstrual period.

The main etiopathology of acute and recurrent pelvic and lower abdominal pain in an older child and adolescent includes gastrointestinal mucosal involvement such as acute gastroenteritis, inflammatory bowel disease, distention, and dysmotility (such as occurs with small bowel obstruction, volvulus, or fecal retention). Extraintestinal causes include intermittent renal pelvic obstruction, pyelonephritis, rupture, or hemorrhage into mesenteric cysts[1]. In some instances, peritoneal irritation of an inflamed bowel could lead to subacute pain as occurs in an atypical presentation of appendicitis. The focus in history is on description of symptoms that permit presumptive etiopathological diagnosis (see Table 1).

**TABLE 1**
**Causes of Acute and Subacute Recurrent Lower Abdominal Pain in Older Children and Adolescents**

Gastrointestinal
- Mucosal
  - Inflammatory bowel disease
  - Atypical colitides (eosinophilic gastroenteritis and lymphocytic colitis)
  - Infectious
- Bowel distention or dysmotility
  - Eosinophilic gastroenteritis with serosal involvement
  - Midgut volvulus/adhesions from previous abdominal surgery/diverticulum
  - Functional fecal retention
- Peritoneal irritation
  - Acute appendicitis with or without periappendiceal inflammation
  - Mesenteric lymphadenitis
  - Mesenteric/omental cysts (hemorrhage or rupture)

Extraintestinal
- Acute pancreatitis
- Genitourinary – pyelonephritis, calculi, pelvic ureteral junction obstruction
- Cystitis

Extraabdominal
- Musculoskeletal – inflammatory/infectious arthritis of hip joint
- Inguinal lymphadentitis (suppurative or nonsuppurative)

Evaluation of a child with lower abdominal and pelvic pain starts with eliciting a relevant history. This holds true for a face-to-face encounter as well as a telephone triage[2]. The character, location, frequency of the pain, and presence of associated symptoms provide clues leading to appropriate testing and also to probable etiology. Episodic or cramping pain with variable intensity may suggest bowel distention or stretching. This pain is usually poorly localized and the child may be unable to point to a specific location of the pain. Localized, sharp pain suggests peritoneal irritation. This pain that is worse with movement and accompanied by fever or systemic signs will require immediate evaluation for peritoneal irritation and inflammation. Pain arising from pelvic structures may be felt in the lower abdominal quadrants or may be described as a deep-seated pain. Tenesmus, an intense pain on defecation, may indicate rectal inflammation (Proctitis) or pelvic or perirectal abscess.

Vomiting, although indicative of gastrointestinal origin, may occur with any severe pain. Acute gastroenteritis or food poisoning is a common cause of acute abdominal pain. However, bilious or bloody emesis portends bowel obstruction or mucosal disease. Intermittent volvulus with a previously silent midgut malrotation may present in an older child or adolescent with severe bilious vomiting and abdominal pain. Vomiting with dry heaving or retching occurs in acute pancreatitis or in intense pain such as occurs in biliary colic or adnexal torsion.

Adolescent patients may not report intermittent passage of blood per rectum or per vagina unless asked in a gentle, nonthreatening manner. This is true of difficult or painful defecation. Bright red blood per rectum, on the surface of the stools, indicates fissures (painful) or a hemorrhoid (painless). Passage of blood with cramps indicates colitis (painful cramps) and occasionally polyps. Meckel’s diverticulum causes painless bleeding. Symptomatic Meckel’s diverticulum may occur in young adults[3]. Intermittent crampy abdominal pain, recent onset difficulty with bowel movements, or abdominal distention may indicate subacute intestinal obstruction. Meckel’s diverticulum may cause distal small bowel obstruction without blood in stools. Colitis presents with bloody diarrhea associated with abdominal cramps. It is often not possible clinically to distinguish between infectious colitis and the first presentation of inflammatory bowel disease. Periodic appearance of blood per rectum accompanied by abdominal cramps should prompt questions about concomitant menstrual periods and a diagnosis of endometriosis considered. Difficulty and pain in passing stools and bright red blood per rectum may suggest Solitary Ulcer Rectum Syndrome. The most commonly reported signs/symptoms in this often-undiagnosed disorder are rectal bleeding, mucus per rectum, pain, and constipation. A history of digital evacuation is often present, but may be often not volunteered by an adolescent. Diagnosis is made by endoscopy and stool softeners or bulk laxatives are the first line of therapy[4].

Systemic symptoms such as fever, weight loss, and fatigue may suggest infectious or inflammatory pathology. Weight loss, recurrent oral sores (“fever blisters”), arthralgia, and history of recurrent or nonhealing anal fissures raise suspicion for inflammatory bowel disease.

Infectious causes of cramping abdominal pain are usually associated with diarrhea and include dysenteric stools in *Salmonella, Shigella*, *Yersinia*, and *Camplyobacter* enteritis. Watery diarrhea can occur in *Aeromonas* and other coliform infections. Possible sources of contact with contaminated water or food such as recent camping or drinking from a running stream of water, or similar illness in close contact, are potential clues.

> Physical examination of the adolescent described above showed a pale looking young adolescent with a heart rate of 100. She was in distress because of the pain. She reported a pain 8/10 on the pain scale. Systemic examination reveals generalized abdominal tenderness with rebound tenderness in the right lower quadrant. A fecal occult blood test was positive.

Particular attention in physical examination should be directed to the presence of a right lower-quadrant mass or tenderness. Subacute pain may be a presenting feature of appendicular mass[5]. Suprapubic and lower-quadrant tenderness may also occur in pelvic or adnexal inflammation. A rectal examination is of particular importance. Presence of occult blood may uncover mucosal inflammation or vault tenderness may lead to an eventual diagnosis of pelvic abscess. Rectal vault filled with stools suggests constipation and will immediately establish a cause for encopresis. Perianal fistula, or deep fissures or skin tags with perianal scarring, are very specific findings for Crohn’s disease. Eosinophilic gastroenteritis and lymphocytic colitis are increasingly diagnosed in children and adolescents. Diarrhea, recurrent acute abdominal pain, and weight loss are common presenting features in eosinophilic gastroenteritis[6]. Watery, nonbloody diarrhea in association with abdominal pain in absence of obvious infectious etiology should prompt suspicion for lymphocytic colitis[7]. In small bowel obstruction such as midgut volvulus, abdominal findings may be subtle and include distention, hyperactive bowel sounds, and absence of peritoneal signs in the face of severe abdominal pain and emesis.

Warning symptoms in a child with acute, recurrent abdominal pain include hematemesis, bilious emesis, bloody diarrhea, localized abdominal pain, febrile illness, and weight loss. Physical examination findings such as rebound or point tenderness, abdominal mass, or presence of blood on rectal examination may point to underlying diagnosis. When the cause of pain is unclear in menstruating or sexually active female adolescents, a pelvic examination by an adolescent specialist is advisable. A history of irregular menstrual periods and intermenstrual vaginal bleeding are other obvious indications.

In summary, during history and physical examination, an attempt should be made to seek a probable etiopathologic diagnosis and direct appropriate choice of tests.

## HOW TO CHOOSE AN APPROPRIATE TEST

> Hemogram of the adolescent female described above showed a white cell count of 12,000/cu mm with a left shift and increased band count. MCV was 69. ESR was 31 mm/h and serum iron 11 mg/dl. Stool cultures were sent and an imaging study was obtained.

Abdominal pain and persistent blood in stools, either overt or occult, requires further evaluation. A hemogram and a peripheral smear could indicate the presence of an underlying acute inflammatory process. Peripheral smear and indices may indicate iron deficiency and/or chronic blood loss. Elevated ESR and/or CRP suggest inflammatory gastrointestinal or extraintestinal abdominal disease. Stool cultures will yield positive results in *Salmonella, Shigella, Yersinia,* and *Camplyobacter* infections and other infectious causes. *Clostridium difficile* toxin assay is often overlooked in the community setting. Although it is the most common cause of nonbloody diarrhea in a nosocomial setting, this infection is associated with community infections and outpatient antibiotic usage[8].

> The abdominal pelvic CT scan with contrast showed fat-stranding and possible phlegmon consistent with appendicitis. Small bowel obstruction was also thought likely. A right ovarian cyst was noted.

The choice of imaging study or an endoscopy as an initial test depends on clinical presentation (see Table 2). Imaging studies such as a CT scan are useful when the presentation is acute; the pain is localized, associated with systemic symptoms such as fever and chills, or signs of peritoneal irritation are present. Intra-abdominal sepsis including appendicular abscess and inflammatory mass ("phlegmon") associated with Crohn's disease are typically diagnosed in this manner. A CT scan may be a test of choice in suspected acute pancreatitis, however, abdominal ultrasound remains the diagnostic test of choice for acute abdominal emergencies in pediatric age group. It is a valuable screening tool for diagnosis of cystic lesions, including abscesses, appendicitis in children, and intussusception, as well as adnexal lesions[9]. Plain X-rays of the abdomen will show excessive stool retention in constipation causing abdominal pain or abnormal bowel gas pattern in subacute bowel obstruction. A strong clinical suspicion of small bowel obstruction such as occurs in midgut volvulus demands an emergent upper gastrointestinal contrast study with water-soluble contrast.

Upper and lower endoscopy should be considered in adolescents with persistent abdominal pain and presence of blood in stools. Inflammatory bowel disease, eosinophilic gastroenteritis, bleeding peptic ulcer, and Solitary Rectal Ulcer Syndrome are some examples of endoscopic diagnosis. Mucosal biopsies are indicated even when the endoscopic visual inspection shows normal mucosa[10,11]. Recurrent diarrhea with or without blood abdominal pain is another indication for endoscopy[12]. Atypical colitides such as eosinophilic gastroenteritis and lymphocytic colitis are increasingly diagnosed by endoscopy and both are characterized by fairly typical histopathological features. Because of the large percentage of patients where the cause of the recurrent pain is mostly functional, we have not discussed the newer and more expensive new technologies such as spiral CT, MRI, or virtual colonoscopy because of the low yield in patients with low index of suspicion for serious organic cause.

**TABLE 2**
**Clinical Guide to Choosing Appropriate Test: Imaging Study or Gastrointestinal Endoscopy**

| Clinical Presentation | Probable Diagnosis | Investigation of Choice |
|---|---|---|
| Acute abdominal pain, localized quadrant pain or rebound tenderness, distention with free fluid, renal colic | Acute appendicitis<br>Mesenteric/omental cyst hemorrhage or rupture<br>Atypical Meckel's diverticulum<br>PUJ* obstruction | Ultrasound |
| Generalized pain with rebound tenderness, pain with and systemic signs such as fever, ureteric colic, obese child with suspected appendicitis, abdominal pain with suspected mass | Appendicular abscess<br>Inflammatory mass associated with IBD**<br>Ureteric or urinary bladder calculi<br>Tumors of the gut | Abdominal and pelvic CT scan with oral and intravenous contrast |
| Abdominal pain with diarrhea, bloody stools, painful defecation with tenesmus | IBD<br>Other colitides | Gastrointestinal endoscopy |
| Abdominal pain with bilious emesis, abdominal distention | Intestinal obstruction | Single contrast barium study or CT scan |

* Pelvic ureteral obstruction.

** Inflammatory bowel disease.

## MANAGEMENT

> Intravascular fluids were initially administered as a bolus and later as maintenance. Surgical consultation was obtained. In discussion with the surgical team, a diagnosis of Crohn's disease was thought more likely based on history, physical examination, and the CT scan findings. A surgical exploration was hence deferred. Stool cultures did not yield any pathogens and *C. difficile* toxin assay was negative. She was started on intravenous antibiotics (Ampiciliin, Gentamicin, and Metronidazole) and kept nil by mouth. TPN was started secondary to weight loss. She reported gradual reduction in abdominal pain, diarrhea, and emesis and a weight gain of 4 lb. A repeat CT scan on hospital day 5 showed interval resolution of peritoneal free fluid, but persistent inflammatory mass in the right lower quadrant as well as the ovarian cyst. A colonoscopy performed 4 weeks later showed endoscopic and histopathological features consistent with Crohn's disease.

The therapy may include surgical or medical management and depends on the likely or established diagnosis. Supportive care such as intravascular volume repletion for pain relief is the initial essential step in management. When intestinal distention and dysmotility are present, nasogastric suction and bowel decompression alleviate discomfort and emesis. Severe and cramping pain such as renal colic and pancreatic pain often require narcotic analgesics, but should not be administered without sufficient evidence for the diagnosis. Intravenous broad spectrum antibiotics are required in intra-abdominal sepsis such as appendicular abscess or in presence of an inflammatory mass (phlegmon) associated with Crohn's disease. Antibiotics are not indicated in acute gastroenteritis or with bloody diarrhea with abdominal cramps caused by infectious or inflammatory bowel disease. Surgical exploration is required if findings are suggestive of appendicitis or with small or large bowel obstruction.

The case history described highlights several points of management. The distinction between a "surgical" or a "medical" cause of abdominal pain may not be straightforward and repeated evaluation is often required. The therapy in such a situation is best decided by a combined team approach. Further management and final diagnosis may not be made until later, but will not preclude adequate and satisfactory immediate intervention.

In summary, acute and recurrent abdominal pain in older children and adolescents may be caused by several disease processes (see Table 1). An etiopathologic approach in history and physical examination will not only help differentiate gastrointestinal and extraintestinal causes, but also guide appropriate choice of tests. Choice of imaging tests or endoscopy is based on presentation and probable diagnosis. Findings on the imaging studies should be interpreted in the light of clinical evaluation and impression. A team approach in management is often essential and surgical exploration may need to be either emergent as in perforated appendicitis or midgut volvulus, or after careful and repeated evaluation based on a combination of history, physical examination, and results of imaging studies.

## BIOSKETCHES

**Harohalli Shashidhar, MD,** Assistant Professor of Pediatrics and Chief, Division of Pediatric Gastroenterology, Department of Pediatrics, University of Kentucky, Lexington. He has completed residency training in Pediatrics and fellowship in Pediatric Gastroenterolgy. His special areas of interest are inflammatory bowel disease and functional gastrointestinal disorders. E-mail: hshas2@uky.edu.

**Hatim Omar, MD,** Professor of Pediatrics and Obstetrics and Gynecology and Director of the Section of Adolescent Medicine, Department of Pediatrics, University of Kentucky, Lexington. Dr. Omar has completed residency training in Obstetrics and Gynecology as well as Pediatrics. He has also completed fellowships in Vascular Physiology and Adolescent Medicine. He is the recipient of the Commonwealth of Kentucky Governor's Award for Community Service and Volunteerism and is well known internationally as an authority in Adolescent Medicine and Pediatric and Adolescent Gynecology. E-mail: haomar2@uky.edu.
